# In *cis TP53* and *RAD51C* pathogenic variants may predispose to sebaceous gland carcinomas

**DOI:** 10.1038/s41431-020-00781-x

**Published:** 2020-12-15

**Authors:** Diana Le Duc, Julia Hentschel, Sonja Neuser, Mathias Stiller, Carolin Meier, Elisabeth Jäger, Rami Abou Jamra, Konrad Platzer, Astrid Monecke, Mirjana Ziemer, Aleksander Markovic, Hendrik Bläker, Johannes R. Lemke

**Affiliations:** 1 Institute of Human Genetics, University of Leipzig Medical Center, 04103 Leipzig, Germany; 2 Department of Evolutionary Genetics, Max Planck Institute for Evolutionary Anthropology, 04103 Leipzig, Germany; 3 Institute of Pathology, University of Leipzig Medical Center, 04103 Leipzig, Germany; 4 Department of Endocrinology, Nephrology, and Rheumatology, University of Leipzig Medical Center, 04103 Leipzig, Germany; 5 Department of Dermatology, Venereology and Allergology, University of Leipzig Medical Center, 04103 Leipzig, Germany

**Keywords:** Genetics research, Cancer genetics

## Abstract

Pathogenic variants in *TP53* have been classically thought to cause Li-Fraumeni syndrome (LFS), a cancer predisposition with high risks for various childhood- and adult-onset malignancies. However, increased genetic testing has lately revealed, that pathogenic variant carriers exhibit a broader range of phenotypes and that penetrance may be dependent both on variant type and modifiers. Using next generation sequencing and short tandem repeat analysis, we identified germline pathogenic variants in *TP53* and *RAD51C* located in *cis* on chromosome 17 in a 43-year-old male, who has developed a rare sebaceous gland carcinoma (SGC) but so far no tumors of the LFS spectrum. This course mirrors a *Trp53*-*Rad51c*-double-mutant *cis* mouse-model, which similarly develops SGC, while the characteristic *Trp53*-associated tumor spectrum occurs with significantly lower frequency. Therefore, we propose that co-occurent pathogenic variants in *RAD51C* and *TP53* may predispose to SGC, reminiscent of Muir-Torre syndrome. Further, this report supports the diversity of clinical presentations associated with germline *TP53* alterations, and thus, the proposed expansion of LFS to heritable *TP53*-related cancer syndrome.

## Introduction

Li-Fraumeni syndrome (LFS) is an autosomal dominantly inherited multicancer predisposition covering a spectrum of five core malignancies: breast cancer, soft tissue or bone sarcoma, brain tumors, and adrenocortical carcinoma [1, 2]. The genetic basis of LFS are germline pathogenic variants in *TP53* [1, 3]. Carriers have a 58% risk of developing cancer before age 40 and about 80% before age 70 [4]. Penetrance varies according to age, sex, and variant type [4]. Thus, penetrance is higher in males than in females during childhood and adolescence, but by age 35 the initial male bias is offset by the burden of breast cancer in women [4]. Lifetime risk of developing cancer has been estimated to 70% or higher for men, while women’s risk is close to 100% [4–7]. The predominance of familial cases included in these studies likely leads to an overestimation of the disease penetrance [1]. Thus, estimating the cancer risk for *TP53* variant carriers remains a great challenge [1]. However, the fact that about 20% of carriers detected in a familial context do not develop cancer until the age of 70 years, suggests that additional genetic or nongenetic factors may create an environment that is either promoting, or restricting LFS development. The presence of modulators may also explain the higher than expected frequency of pathogenic *TP53* variants in the general population [8].

In support of genetic modulators of *TP53*, a mouse model harboring a *Trp53*-null-allele and a *Rad51c*-null-allele displayed different phenotypes depending on the location of the mutant alleles (on the same (*cis*) or on the alternate mouse chromosome 11 (*trans*)). *Trans* mice developed tumors with latency and spectrum similar to LFS, while *cis* mice had sebaceous glands carcinoma (SGC) and developed fewer tumors characteristic of *Trp53*-null-allele [9] (for a detailed description of the mouse model and its limitations see Supplementary Material).

Germline pathogenic variants in *RAD51C* predispose to hereditary breast and ovarian cancer [10, 11], and, to our knowledge, SGC does not belong to the tumor spectrum. SGC is frequently observed in the context of Muir-Torre syndrome (MTS) due to mismatch repair (MMR) deficiency related to hereditary non-polyposis colorectal carcinoma syndrome [12], but do not belong to the typical LFS-related tumor spectrum. To our knowledge SGC has been previously described in only one patient with a pathogenic *TP53* variant (c.818G>A, p.(Arg273His)) [13]; in this case, it remains unclear whether pathogenic variants affecting other genes co-occurred.

**Fig. 1:**
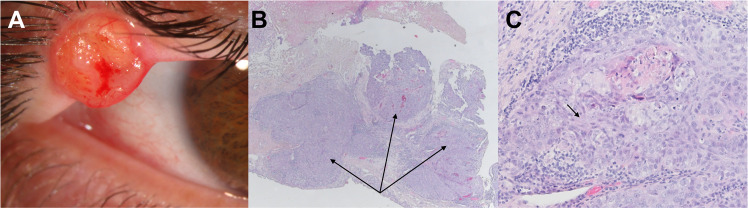
Presentation of the sebaceous gland carcinoma. **A** Clinical appearance of the sebaceous gland carcinoma as a right upper lid mass. **B** Hematoxylin eosin staining of the resected mass (10×). The arrow indicates irregular lobules and sheets of atypical sebaceous cells. **C** Hematoxylin eosin staining of the resected mass (20×). The arrows indicate undifferentiated, atypical cells with considerable nuclear pleomorphism and eosinophilic cytoplasm.

We present a patient that harbors a pathogenic missense variant in *TP53* located in *cis* with a deletion within *RAD51C* on human chromosome 17. Based on the IARC *TP53* [14] database about 71% of the pathogenic variations are missense. Our patient is a 43-year-old male, who was diagnosed with a mid-occipital basal cell carcinoma (BCC) at age 38. At the age of 41, a right upper lid SGC was diagnosed. Both tumors had been fully excised shortly after diagnosis and the further clinical course was uneventful without any additional treatment necessary. Apart from colon cancer of the maternal grandmother at >80 years, family history is negative for any tumor diseases. This is not unexpected since 7–20% of the *TP53* variants occur de novo [15, 16], and hence tumor family history is absent. The proband has two healthy 4 year-old twin children of different gender (Fig. 1C).

Although our observations are limited given the LFS known penetrance of around 58% at the age of 40 [4], the proband appears to recapitulate the mouse phenotype with no LFS typical tumors, but SGC reminiscent of MTS.

## Methods

### Histologic examination

An initial biopsy of the tumor followed by hematoxylin and eosin staining showed a malignant epithelial tumor containing basophilic sebaceous tumor cells with a nodular and in part trabecular order and no peripheral palisading.

### Molecular and bioinformatic analyses

Genomic DNA was extracted from peripheral blood of the proband and his relatives, and, to determine whether the variant is of germline-origin or mosaicism, in the proband also from finger nails. We performed panel sequencing (Illumina TruSight Cancer Panel) targeting 94 cancer-related genes (Table S1 in the Supplementary Material) on a NextSeq500/550 High Output platform. Coverage of at least 20-fold was obtained for 99.4% of the target sequences. We evaluated data using Varvis software (Limbus, Rostock). Copy number variations (CNV) were analyzed using the coverage information of the panel with the current version of the Varfeed CNV software. The deletion of exons 5–9 in *RAD51C* was validated by Multiplex Ligation-dependent Probe Amplification (MLPA) (Kit P260, MRC-Holland) and the *TP53* variant was validated by Sanger sequencing.

To investigate possible recombination events we performed a short tandem repeat (STR) analysis in the proband, his parents, and his children using loci located on chromosome 17 (Table S2 in the Supplementary Material).

To assess a potential loss of heterozygosity (LOH) in the tumor, we performed the same panel sequencing. The proportion of normal tissue within the tumor section was approx. 30%. Of the target sequences 99.9% were covered at least 20 times. Variant calling was performed with Strelka2 [17], to increase sensitivity. We used samtools [18] to determine the read depth. For a detailed description of the methods, see the Supplementary Material.

## Results

### Diagnostic evaluation

Histological examination of the resected tumor established the diagnosis of an SGC (Fig. 1A–C).

**Fig. 2:**
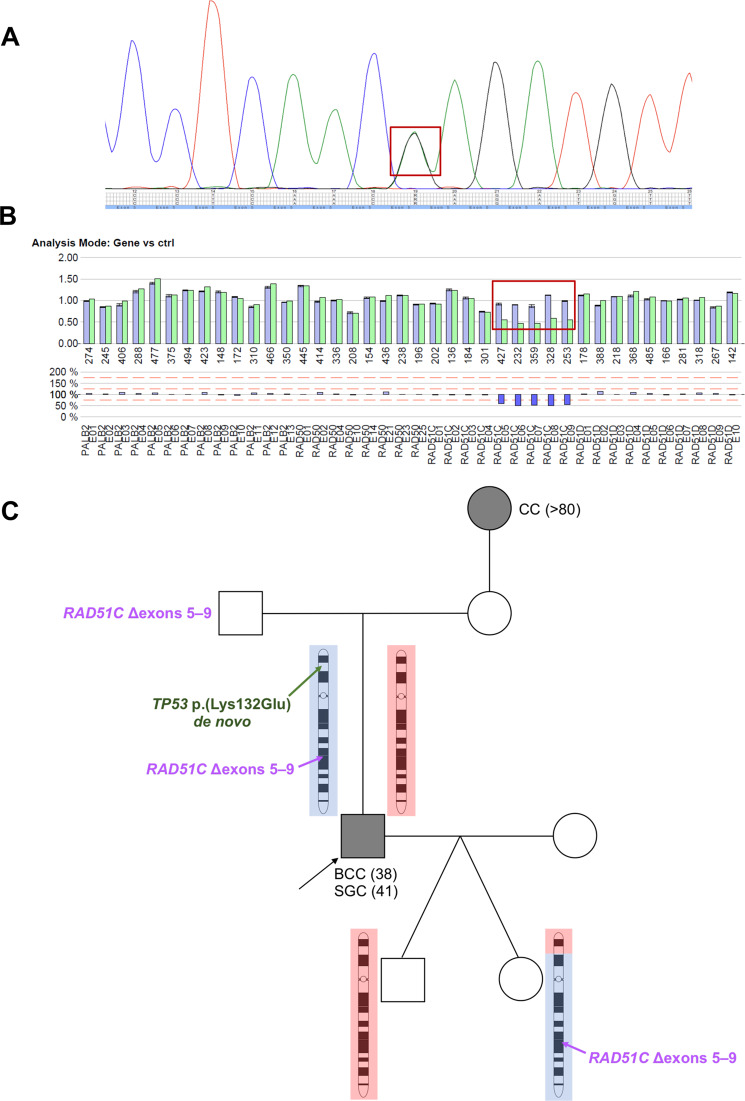
Identified pathogenic variants. **A** Electropherogram of the Sanger sequencing and the heterozygous NM_000546.5, c.394A>G *TP53* variant (marked in the red quadrant). **B** MLPA validation of the exons 5–9 deletion, NM_058216.2 in *RAD51C* (marked in the red quadrant). **C** Pedigree chart of the family: the proband is indicated with an arrow. The origin of the paternal (blue) and maternal (red) chromosome has been established based on STR markers length (Fig. S4 in Supplementary Material). The son inherited the chromosome originating from his paternal grandmother, without recombination, and with wild type alleles of *TP53* and *RAD51C*. The daughter inherited a recombined chromosome 17 with the beginning of p arm originating from the paternal grandmother, with a wild type *TP53* allele, and the rest originating from the paternal grandfather, harboring the deletion within *RAD51C*. Location of the de novo mutated *TP53* allele (in green) on the paternal originating chromosome of the proband, thus situated in *cis* with the deletion within *RAD51C*. CC colon carcinoma, BCC basal cell carcinoma, SGC sebaceous gland carcinoma, age at diagnosis in years is given between brackets.

In line with the normal MMR protein expression patterns (Fig. S1 in the Supplementary Material), we did not detect any sequence alteration in MMR genes by panel sequencing of DNA from blood and tumor. However, we detected the heterozygous missense germline variant chr17:7,578,536; NM_000546.5, c.394A>G, p.(Lys132Glu) in *TP53*, which was classified as pathogenic [19] (Supplementary Material). The variant is located in the DNA-binding domain of p53 and functional examination in yeast revealed a dominant negative (DN) effect of the altered allele with a mean residual activity of 1% or less compared to wild-type [20, 21]. The variant has been described in the context of Li-Fraumeni [21, 22] in one pedigree and has been recently classified as a true recurrent pathogenic LFS variant [8] (for phenotype description and variant classification see Supplementary Material). CNV analysis of the proband further revealed a deletion of exons 5–9 of *RAD51C* (NM_058216.2, c.(235 + 1_236-1)_(*120_?)del), located also on chromosome 17. This deletion has already been described as pathogenic in the context of familial breast and ovarian cancer [23–25] (for phenotype description and variant classification see Supplementary Material). Both variants were validated using a second detection method (Fig. 2A, B) and in an additional tissue (Figs. S2A and S3A in the Supplementary Material). No further clinically relevant variants could be identified in cancer-relevant genes using the above panel.

### Chromosomal localization of the variants

Segregation analysis in the proband’s family revealed that the deletion within *RAD51C* is paternally inherited, while the *TP53* variant occurred de novo (Pedigree in Figs. 2C and S2 in the Supplementary Material). Phasing of the two variants was done using STR analysis (Fig. S4 in the Supplementary Material). We initially excluded the *TP53* variant in both children of the proband (Figs. 2C and S2D–E in the Supplementary Material). Using STR analysis we show that the daughter inherited a recombined chromosome 17, while there was no evidence for a crossing-over event in the son (Figs. 2C and S4, Table S3 in the Supplementary Appendix). In accordance with the STR analysis, we confirmed the deletion within *RAD51C* in the daughter (Figs. 2C and S3 in the Supplementary Material). We evaluated the probability of recombination for the *TP53* and *RAD51C* variants to be 31.6%, using $d = 50{\mathrm{ln}}( {\frac{1}{{1 - 2{\rm{Pr}}[ {{\rm{recombination}}} ]}}})$, where *d* represents the distance in centimorgans (the two genes are at ca. 51 × 10^6^ bp apart corresponding roughly to 51 centimorgans [26]). Thus, the *TP53* variant occurred de novo on the paternal chromosome of the proband, and thereafter in *cis* with the deletion within *RAD51C* (Fig. S4 in the Supplementary Material).

## Discussion

Here, we describe a proband with a de novo pathogenic *TP53* variant associated with an in *cis* inherited pathogenic deletion within *RAD51C* (Fig. 2C). Since the *TP53* variant occurred de novo the family history for LFS was absent. It is remarkable that, although the proband harbors a variant of severe deficiency with a dominant negative effect, known to significantly increase cancer risk [21], no LFS-typical tumors occurred until his current age of 43 years. This is in high accordance with a *Trp53*-*Rad51c*-double-mutant mouse model, which carries the mutant alleles in *cis* [9]. The *Rad51c* loss promoted SGCs and skin malignancies, but reduced tumors characteristic of *Trp53*-mutant mice [9]. The human proband also developed SGC and a BCC, recapitulating the mouse phenotype.

To date, MTS due to dysfunctional MMR is the only known autosomal dominant mendelian condition predisposing to SGC. In both our proband and the mouse model, [9] MMR was intact (Fig. 1D–F), suggesting an alternative mechanism for these tumors. Indeed, p53 dysfunction was previously suggested to be a divergent pathway in the molecular pathogenesis of SGC that show strong nuclear p53 staining and intact MMR [27].

To our knowledge, SGC has been described in only one patient with a pathogenic *TP53* variant [13]. It remains unclear whether this individual had either typical LFS as well as an additional predisposition to SGC, or potentially an underlying genetic disorder similar to the mechanism described in this manuscript (no information is available on sequence alterations of *RAD51C* in this published individual). Although further studies are required to validate and fully elucidate the molecular mechanism, our observations point towards two major clinical implications: (1) SGC could be related to co-occurrence of pathogenic *TP53* and *RAD51C* variants, and cause a phenotype reminiscent of MTS independent of MMR deficiency. Moreover, *TP53* was found to harbor the highest number of pathogenic variants in a set of SGCs [28], suggesting that germline pathogenic variants in *TP53* potentially associated with other modifiers may be more frequent than expected. (2) In line with the observations in *Trp53*-*Rad51c*-double mutant *cis* mice [9], also human in *cis* co-occurrence of pathogenic *TP53* and *RAD51C* variants may substantially transform the Li-Fraumeni phenotype to a predisposition to SGC; however, this may not preclude the development of LFS typical tumors.

In order to establish a clear genotype–phenotype correlation future patients with co-occurrence of *TP53* and *RAD51C* pathogenic variants, additional to the mouse model are needed. Thus, our recommendation for the present patient was to undergo regular LFS screening [1] (for ethical considerations and patient management see Supplementary Material). Lately, multiple studies have demonstrated the phenotypic variability of *TP53* pathogenic variants carriers [29, 30]. This has been related to both variant type and potential modifiers [1, 29, 30]. Bougeard and colleagues suggested that a clinical gradient can be identified in *TP53* pathogenic variants carriers, depending on the variant type. Hence, they suggested that future studies should characterize genotype–phenotype correlations and modifiers of the phenotype, such that patients could benefit from a stratified clinical management [29]. Our brief report adds to the heterogeneity of the heritable *TP53*-related cancers and aims to raise awareness on potential modifiers. If this is confirmed by other studies a clinical management stratification could be implemented to the benefit of such patients.

## Database submissions

Variants have been submitted to ClinVar (SUB8180034): https://www.ncbi.nlm.nih.gov/clinvar/

- *TP53*: NM_000546.5:c.394A>G, SCV001429318;
- *RAD51C*: NM_058216.2:c.(235 + 1_236-1)_(*120_?)del, SCV001438810.

## Supplementary information

Supplementary Appendix
