# Supplementary material for: In cis TP53 and RAD51C pathogenic variants may predispose to sebaceous gland carcinomas
Source: Eur J Hum Genet. 2020 Dec 15;29(3):489–94. doi: 10.1038/s41431-020-00781-x (PMC7940394; doi:10.1038/s41431-020-00781-x)
Supplement: Supplementary file 1 — Supplementary Appendix [file 41431_2020_781_MOESM1_ESM.docx]

**Supplementary Appendix for**

**In *cis* *TP53* and *RAD51C* pathogenic variants may predispose to sebaceous gland carcinomas**

Diana Le Duc ^1, 2*^ MD/PhD, Julia Hentschel ^1^ PhD, Sonja Neuser ^1^ MD, Mathias Stiller ^1, 3^ PhD, Carolin Meier ^1^ MSc, Elisabeth Jäger ^4^ PhD, Rami Abou Jamra ^1^ MD, Konrad Platzer ^1^ MD, Astrid Monecke ^3^ MD, Mirjana Ziemer ^5^ MD, Aleksander Markovic ^5^ MD, Hendrik Bläker ^3^ MD, Johannes R. Lemke ^1*^ MD

Running Title: *Cis* *TP53* and *RAD51C* pathogenic variants cause SGC

1 Institute of Human Genetics, University Medical Center Leipzig, Leipzig, 04103, Germany

2 Department of Evolutionary Genetics, Max Planck Institute for Evolutionary Anthropology, Leipzig, 04103, Germany

3 Institute of Pathology, University Medical Center Leipzig, Leipzig, 04103, Germany

4 Department of Endocrinology, Nephrology, and Rheumatology, University Medical Center Leipzig, Leipzig, 04103, Germany

5 Department of Dermatology, Venereology and Allergology, University of Leipzig Medical Center, Leipzig, 04103 , Germany

***Correspondence to:**

Johannes Lemke

Phone: +49 (0)341 97 23800

Fax: +49 (0)341 97 23819

Email: [johannes.lemke@medizin.uni-leipzig.de](mailto:johannes.lemke@medizin.uni-leipzig.de)

Diana Le Duc

Phone: +49 (0)341 97 23800

Fax: +49 (0)341 97 23819

Email: [gabriela-diana.leduc@medizin.uni-leipzig.de](mailto:gabriela-diana.leduc@medizin.uni-leipzig.de)

[diana_leduc@eva.mpg.de](mailto:diana_leduc@eva.mpg.de)

**TABLE OF CONTENTS**

[Supplementary Methods 3](#_Toc55127111)

[HISTOLOGIC DIAGNOSIS AND IMMUNOHISTOCHEMICAL STAINING 3](#_Toc55127112)

[DNA EXTRACTION 3](#_Toc55127113)

[TRUSIGHT CANCER SEQUENCING PANEL AND DATA ANALYSIS 4](#_Toc55127114)

[SANGER SEQUENCING 4](#_Toc55127115)

[MULTIPLEX LIGATION-DEPENDENT PROBE AMPLIFICATION (MLPA) 5](#_Toc55127116)

[SHORT TANDEM REPEAT (STR) ANALYSIS 5](#_Toc55127117)

[Supplementary Text 6](#_Toc55127118)

[VARIANT CHARACTERIZATION 6](#_Toc55127119)

[ETHICAL CONSIDERATIONS AND PATIENT MANAGEMENT 7](#_Toc55127120)

[MOUSE MODEL 9](#_Toc55127121)

[PRESENCE OF GERMLINE VARIANTS IN TUMOR 10](#_Toc55127122)

[Supplementary Figures 11](#_Toc55127123)

[Figure S1. 11](#_Toc55127124)

[Figure S2. 12](#_Toc55127125)

[Figure S3. 13](#_Toc55127126)

[Figure S4. 14](#_Toc55127127)

[Figure S5. 15](file:////Users/diana_leduc/Documents/TP53/Manuscript/EJHG/Revision2/LeDuc_et_al_TP53-RAD51C_Supplementary_Appendix_R2.docx#_Toc55127128)

[Supplementary Tables 16](#_Toc55127129)

[Table S1. 16](#_Toc55127130)

[Table S2. 17](#_Toc55127131)

[Table S3. 18](#_Toc55127132)

[Supplementary References 19](#_Toc55127133)

# Supplementary Methods

## HISTOLOGIC DIAGNOSIS AND IMMUNOHISTOCHEMICAL STAINING

Initially a biopsy from the right upper eyelid was taken. Hematoxylin and eosin (H&E) morphology showed a malign epithelial tumor with basophilic sebaceous tumor cells with a nodular and in part trabecular order with no peripheral palisading. The biopsy was followed by complete resection of the tumor. The immunohistochemical phenotype was CK8+, CK7+, p63+, BerEP4- with a proliferation of 45% (MIB-1).

Immunohistochemical staining for DNA mismatch repair proteins was performed on formalin-fixed, paraffin embedded tissue sections using a Ventana BenchMark Ultra with antibodies against MSH2 (G219-1129), PMS2 (A16-4), MLH1 (M1), MSH6 (SP93) from Ventana. For p53-staining the monoclonal mouse antibody from Dako (DO-7) was used. Staining protocols after deparaffinization were as follows:

| **Antibody** | **Pretreatment / Duration** | **Dilution** | **Detection Kit** |
| --- | --- | --- | --- |
| MLH1 | Cell Conditioning 1 (CC1) / 80' | Prediluted | OptiView DAB |
| MSH2 | CC1 / 40' | Prediluted | OptiView DAB |
| PMS2 | CC1 / 88' | Prediluted | OptiView DAB |
| MSH6 | CC1 / 64' | Prediluted | OptiView DAB |
| p53 | CC1 / 36' | 1:100 | UltraView DAB |

Hematoxylin was used for counterstaining.

## DNA EXTRACTION

Genomic DNA was isolated from 400µl EDTA^[[1]](#footnote-1)^ blood via an automated processing method using the MagCore® HF16 Plus and the MagCore® Genomic DNA Whole Blood Kit following the manufacturer’s instructions. In brief, the sample homogenate is lysed using proteinase K, followed by addition of cellulose coated magnetic beads to capture the DNA, which is finally eluted in 100µl TE buffer ^[[2]](#footnote-2)^. DNA concentration was initially measured using a NanoDrop™ 2000/2000c spectralphotometer, followed by Qubit dsDNA High-Sensitivity (ThermoFisher Scientific™, Massachusets, U.S.A.).

Genomic DNA from nails was extracted using QIAamp® DNA Mini Kit (Qiagen, Minden, Germany) following the user developed protocol ^[[3]](#footnote-3)^.

Tumor DNA was extracted using the QIAamp® DNA Micro kit (Qiagen, Minden, Germany), a standard column-based procedure, following manufacturer’s instructions. The amount of contamination with normal DNA was assessed to ca. 30% based on microscopical examination.

## TRUSIGHT CANCER SEQUENCING PANEL AND DATA ANALYSIS

DNA libraries were prepared following the manufacturer’s instructions (TruSight® Cancer Sequencing Panel, Illumina, California, U.S.A.). Briefly, 50ng DNA from the proband (genomic DNA from blood or tumoral DNA) was fragmented to 300bp^[[4]](#footnote-4)^, followed by indexing and PCR amplification. Pooled libraries were then hybridized onto TruSight Cancer probes, followed by enrichment using streptavidin coated magnetic beads and purification. 24 pooled libraries were sequenced 2 × 150bp paired-end reads via NextSeq 500/550 Mid Output v2 kit (300 cycles) (Illumina, California, U.S.A.); Sequencer: Illumina NextSeq 500/550.

Data was processed using the Varvis® pipeline (Limbus Medical Technologies, Rostock, Germany). For the tumor sample, we additionally performed variant calling using strelka-2.9.2, which incorporates a sample contamination model and offers an improved analysis for tumor samples ^1^. We filtered variants with bcftools ^2^ and coverage was assessed using samtools depth ^2^. Data was processed in R ^3^ to assess loss of heterozygosity and coverage using the following packages: dpylr ^4^, ggplot2 ^5^, reshape2 ^6^, and car ^7^.

## SANGER SEQUENCING

Sanger Sequencing was performed for validation and segregation purposes using genomic DNA from proband’s blood or nails, or genomic DNA from blood for proband’s parents and children. We additionally performed sequencing on DNA extracted from the buccal swab of the daughter to exclude mosaicism. An initial PCR was set using the Qiagen® Maxima Hot Start Taq Kit (Minden, Germany) and the following PCR primers (*TP53*, Ex 5+6 F: cacttgtgccctgactttca; *TP53*, Ex 5+6 R ttgcacatctcatggggtta), according to the manufacturer’s instructions. Cleanup of the PCR product was performed with ExoSAP-IT™ (ThermoFisher Scientific™, Germany). The product was sequenced using BigDye™ Terminator v3.1 Cycle Sequencing Kit (ThermoFisher Scientific™, Germany). Electrophoresis was done on an Applied Biosystems® Sanger Sequencing 3500 Analyzer (ABI3500). We used the SeqPatient module of SeqPilot64 (JSI Medical, Ettenheim, Germany) to analyze the data.

## MULTIPLEX LIGATION-DEPENDENT PROBE AMPLIFICATION (MLPA)

We used the SALSA MLPA P260 PALB2-RAD50-RAD51C-RAD51D probemix version 260-B1 (MRC-Holland, Amsterdam, the Netherlands) to validate and segregate the *RAD51C* deletion of exons 5–9. Validation was performed on genomic DNA extracted from proband’s blood and nails. To segregate the deletion we tested the proband’s parents, children, and partner to exclude the possibility that the variant observed in children originates from the proband’s partner. We additionally performed sequencing on DNA extracted from the buccal swab of the daughter to exclude mosaicism. PCR products were separated on a ABI3500 with a dilution 1:60 in HiDi formamide an LIZ600 standard. We analyzed the data using the MLPA module of SeqPilot64 (JSI Medical, Ettenheim, Germany).

## SHORT TANDEM REPEAT (STR) ANALYSIS

To assess the event of meiotic recombination in the proband we performed STR analysis of chromosome 17 using markers located around the *TP53* and *RAD51C* chromosomal location (Tables S2 and S3, Figure S4). We initially performed a Multiplex PCR using the Qiagen Kit (Minden, Germany) followed by capillary electrophoresis on a ABI3500 and GeneMapper analysis.

# Supplementary Text

## VARIANT CHARACTERIZATION

Using the TruSight Cancer Sequenicing Panel, which includes genes that are presently known to confer an increased predisposition to cancer (Table S1), we detected the heterozygous variant chr17:7578536; NM_000546.5, **c.394A>G, p.(Lys132Glu) in *TP53*** (exons are numbered like in NM_000546.5, NG_017013.2 which starts with exon 1 and ends with exon 11; exons are consecutively numbered). The variant occurs once in the general population (GnomAD ^8^), however a recent curation of *TP53* variants that occur in general population, evaluated the variant as pathogenic ^9^. Moreover, this study shows that pathogenic *TP53* germline variants occur in general population more often than previously suspected ^9^. Thus, general population databases should be used with caution in the assessment of oncogenic variants. In ClinVar ^10^ (Variation ID: 376626), there are two assessments of the variant occurring in germline: the first one from 2016 assessed the variant with uncertain significance in respect to Li-Fraumeni syndrome. The evaluator acknowledged that the variant had been described in a Li-Fraumeni family ^11^ and that multiple functional studies have proven an alteration of the normal p53 functions in cell cycle and apoptosis control as a result of the Lys132Glu exchange ^11-13^. Yet, the relevance for the phenotype was deemed as uncertain, since the variant appears in one person in the general population. In 2018 the variant was found in an additional individual. A renewed evaluation assessed the variant as likely pathogenic in respect to hereditary cancer predisposing syndrome. Furthermore, the recent curation of *TP53* pathogenic variants established the pathogenicity of the c.394A>G, p.(Lys132Glu) in *TP53* based on the functional studies and four different non-overlapping datasets of *TP53* pathogenic variants ^9^.

Thus, based on the ACMG^[[5]](#footnote-5)^ criteria ^14^ we evaluated the chr17:7578536; NM_000546.5, c.394A>G, p.(Lys132Glu) variant in *TP53* as pathogenic: PS3 – included several functional studies, which proved the severe deficiency, dominant negative effect of the variant ^11-13,15^, PS4 (moderate) – based on ClinVar, two additional Li-Fraumeni families from literature ^11-13,15^ and GnomAD frequency, the prevalence in affected individuals is higher than in controls, PM5 – two additional changes at position 132: Lys132Arg and Lys132Gln are described as pathogenic ^11-13,15^ , and PP3 – *in silico* the variant is predicted to be damaging ^16,17^.

The only clinical information for a pedigree with c.394A>G, p.(Lys132Glu) variant in *TP53* is available from Goi *et al*. ^11^: the index is a boy who developed an rhabdomyosarcoma at age 2 and an osteosarcoma at 11 years, his mother and his maternal grandmother, also carriers of the variant, developed breast cancer, and his carrier brother was healthy at age 34. There is no clinical information available from the two submissions present in ClinVar; however, both of them relate to hereditary cancer syndromes.

We further detected a **deletion of exons 5–9 of *RAD51C*** (NM_058216.2, c.(235+1_236-1)_(*120_?)del) (exons are numbered like in LRG_314t1 (NM_058216.1), NG_023199.1 which starts with exon 1 and ends with exon 9; exons are consecutively numbered). This deletion has already been described as pathogenic in respect to breast and ovarial cancer ^18-20^ and since it is a truncating variant, we also evaluated it as pathogenic ^14^: PVS1 – null variant multiexon deletion in *RAD51C*, where loss-of-function is a known mechanism of disease ^18-20^, PM2 – absent from controls. The deletion of exons 5–9 most probably leads to an expressed truncated transcript, since exon 4 ends in phase and blood-derived *RAD51C* transcripts from a carrier of the deletion showed normal levels of amplicons spanning the first 4 exons, but significantly lower levels of the amplicons spanning exons 7–9 ^19^.

Clinical information of the *RAD51C* deletion of exons 5–9 is available from three families. In one family the index developed an invasive ductal breast carcinoma at age 41; however, no additional data is available for this family ^20^. In another family the index was affected by early-onset and bilateral invasive ductal breast carcinoma (age 33 years, age 39 years). The deletion was inherited from the mother who developed colon cancer at age 44. The maternal grandmother developed B-cell lymphoma at age 78 and the sister of the maternal grandmother suffered from breast carcinoma at age 55 ^20^. In the third family the deletion was identified in dizygotic twins, one of which was affected by early-onset invasive ductal breast carcinoma (age 42 years) and one by early-onset ovarian carcinoma (age 43 years). Carcinomas were not reported in any other family members and it remains unclear whether in this case the deletion was inherited or occurred *de novo* ^20^.

## ETHICAL CONSIDERATIONS AND PATIENT MANAGEMENT

Genetic counselling of the patient and his family comprised issues regarding surveillance recommendations, genotype-phenotype correlation, risk to family members, and ethical considerations for predictive testing. After identification of the pathogenic variants in *TP53* and *RAD51C*, we informed the patient about the risks of developing cancers of the Li-Fraumeni syndrome (LFS) spectrum and ^21,22^ – in case of female carriers in the family – risk for *RAD51C*-associated hereditary breast and ovarian cancer ^23-25^. We recommended the surveillance program for patients with LFS. At that time, no national guideline for LFS was available, so we used the National Comprehensive Cancer Network (NCCN) guidelines ^26^. Updated European guidelines ^21^ were published in May 2020, though they do not differ remarkably from the NCCN guidelines. Given the unremarkable family history for carcinomas, it was not possible to adapt the recommendations individually. Furthermore, we discussed with the patient the observations of the mouse model in a research context. We underlined that these findings cannot be transferred to supervision or treatment of his case. No (additional) surveillance program can be recommended for a possible risk elevation for recurrent sebaceous glands carcinoma, but LFS surveillance program covers cutaneous malignomas in general (annual clinical examination). Predictive testing of his parents regarding both pathogenic variants was offered, which would also imply a segregation analysis confirming whether the variants occur in *cis* or in *trans*. The parents consented and targeted testing was performed. In a second appointment, together with his parents, we explained the *de novo* origin of the *TP53* variant and the resulting recurrence risk of around 1 % for the patient’s brother (potential germline mosaicism of the parents), for whom predictive testing of both variants can be performed. The unremarkable family history for LFS cancers was explained by the *de novo* origin of the pathogenic *TP53* variant. The patient’s father was confirmed as carrier of the *RAD51C* variant, so no further surveillance advice regarding *RAD51C* was necessary for the parents.

Predictive testing of children regarding LFS should be systematically offered following current recommendations (Rec. 7 with pathogenic dominant-negative missense variant ^26^). The patient and his wife consented for predictive testing for LFS of both children after the counselling regarding cancer risks. If they were tested positive for the variant, surveillance program would have started immediately.

On a research basis we explained to the index patient that based on short tandem repeat (STR) markers we can determine for his children which chromosome was inherited from him (originating from the paternal grandfather or grandmother). However, since the grandfather is carrier of the *RAD51C* variant, this analysis will include as incidental finding the carrier status for *RAD51C*. Thus, within the informed consent the parents were asked whether incidental findings should be communicated. Based on the European Society of Medical Oncology *RAD51C* is a high actionability cancer susceptibility gene ^23^ that is under current review for inclusion in the American College of Medical Genetics secondary findings gene list. Since parents wanted to have secondary findings reported, we further tested the *RAD51C* in support of the linkage analysis. *RAD51C* variant was excluded in the son, whilst the daughter harboured the deletion within *RAD51C*, in support of the linkage analysis result. To exclude any confounding information and since *RAD51C* result could have been interpreted as important for the index’ partner (if linkage information is missing), we additionally tested the partner of the index. The daughter of the index will be offered renewed genetic counselling and surveillance program for *RAD51C*-associated hereditary breast and ovarian cancer (currently starting at the age of 30 ^27^ according to the recommendations of the German Consortium for familial breast and ovarian cancer).

## MOUSE MODEL

In a mouse model aiming to understand the relevance of *Rad51c* in tumorigenesis, Kuznetsov *et al*. initially showed that a *Rad51c* knock-out mouse is not viable, with death occurring in the embryonic stage ^28^. However, lethality could be delayed on a *Trp53*-null background. *Rad51c* and *Trp53* are both located on the chromosome pair 11 in mouse, thus the authors further inquired a heterozygous deficient mouse model for both genes. If both mutant alleles resided on the same chromosome the mice were deemed *cis*, or *trans* if each mutant allele resided on a different chromosome.

The authors studied 5 mouse cohorts over the course of 600 days. Sick mice, or those with visible tumors were sacrificed before term. Interestingly *trans* mice developed tumors in general with the same spectrum and latency as the *Trp53*^ko/+^ mice. The median tumor free survival time: for *Trp53*^ko/+^ females was 475 days *vs.* males 385 (*p*-value not significant); for *trans* females it was 461 days *vs.* males 334 (*p*-value = 0.001); for *cis* females it was 360 days *vs.* males 437 (*p*-value = 0.05). Thus, *trans* males developed tumors earlier than females of the same genotype, while the correlation was reversed for *cis* mice.

Additionally, *trans* mice showed the same tumor spectrum as *Trp53*^ko/+^, mainly dominated by osteosarcomas and muscle sarcomas with only 15% of tumors represented by SGCs. However, only 25% of the *cis* males developed sarcomas and SGCs were much more frequent: 10 of 24 animals (42%) developed preputial gland, 5 (21%) Zymbal’s gland, and 5 (21%) muzzle area carcinomas. The authors claimed that the shift from aggressive *Trp53*-characteristic tumor types to preputial and Zymbal's gland carcinomas could be the reason for longer survival in the *cis* group. Yet, *cis* females usually developed mammary gland carcinomas and carcinomas of the skin and nasal malignancies, which imposed their sacrifice earlier. This was further supported by an additional study, which knocked-out the two genes specifically in epidermal tissues, and showed that the additional loss of Rad51c shortens the latency of p53-deficient mouse tumors ^29^.

In respect to the sebaceous gland tumor characteristics, the authors inquired the microsatellite instability and Msh2 expression. Both analyses showed that a defect in mismatch repair is not responsible for sebaceous tumors in *cis* mice. Furthermore, they noted that tumors from *Trp53*^ko/+^ mice lost the wild-type copy of the *Trp53*. Tumors from trans mice lost the wild-type copy of *Trp53* and retained only the wild-type *Rad51c*, being thus genotypically equal to tumors from *Trp53*^ko/+^ mice. However, in *cis* mice both wild-types alleles of *Trp53* and *Rad51c* got lost in tumors, such that only the non-functional alleles were retained. *In vitro* analyses of mouse embryonic fibroblast cells (MEFs) further showed that cells that are functionally null for both *Rad51c* and *Trp53* (like tumors from *cis* mice) are genetically more unstable than *Trp53*^ko/ko^ MEFs (corresponding to *trans* or *Trp53*^ko/+^ mice). The authors suggest that this may influence the tissue specificity of the tumors.

## PRESENCE OF GERMLINE VARIANTS IN TUMOR

In the mouse *Rad51c*^-/+^/*Trp53*^-/+^ *cis* model it was suggested that the loss of Rad51c wild-type allele from tumor overrides the tissue-specific effect of *Trp53* mutation and promotes a shift from sarcomas to malignancies of skin and adnexa, especially of specialized sebaceous glands ^30^. We thus, confirmed that the proband’s tumor exhibits a similar pattern. CNV analysis revealed a complete loss of exons 5–9 in *RAD51C* and only one copy of exons 1–4. Hence, similar to mice, the *RAD51C* wild-type allele is lost (Figure S5A). However, the *TP53* variant did not show LOH (alternative allele frequency in the tumor was 0.61 *vs*. 0.48 blood, Figure S5B in the Supplementary Material). The absence of LOH was further supported by immunohistochemical staining of p53 in the tumor, which showed a rather increased expression (Figure S5C).

While in the mouse SGC *Trp53* wild-type is also lost, in the human SGC we do not observe LOH, and staining is suggestive for a rather increased expression of p53 (Figure S5C). This may be explained by the fact that the mouse harbors a *Trp53* knock-out allele, while the human proband carries a DN variant. It was shown in a mouse model that DN activity of mutant p53 shortened survival when mutant p53 was highly over-expressed compared to the level of endogenous, wild-type protein ^31^, suggesting thus an advantage for the tumor, similar to LOH for the null alleles.

# Supplementary Figures

Figure S1. **Immunohistochemical staining of the sebaceous gland carcinoma.** Panel A shows MSH2-staining of the tumor (5 ×). Panel B shows MSH6-staining of the tumor (2.5 ×). Panel C shows PMS2-staining of the tumor (2.5 ×). Panel D shows MLH1- staining of the tumor (2.5 ×). Arrows indicate the tumor tissue

Figure S2. **Electropherogram of the Sanger sequencing for the locus chr17:7578536; NM_000546.5, c.394 in *TP53* (marked in the red quadrant).** Panel A shows the proband (genomic DNA extracted from nails) with the variation NM_000546.5, c.394A>G. For the other family members we used genomic DNA extracted from blood for sequencing. Panel B: proband’s mother. Panel C: proband’s father. Panel D: proband’s daughter. Panel E: proband’s son. None of the other family members carries the *TP53* variant, which occurred *de novo* in the proband.

Figure S3. **MLPA P260 PALB2-RAD50-RAD51C-RAD51D results.** Panel A shows the proband (genomic DNA extracted from nails). For the other family members we used genomic DNA extracted from blood for the MLPA analysis. Panel B: proband’s mother. Panel C: proband’s father. Panel D: proband’s daughter. Panel E: proband’s son. Panel F: proband’s partner, to exclude the possibility that the proband’s daughter inherited the variant from her mother. The *RAD51C* deletion of exons 5–9 (in the red quadrant) is inherited by the proband from his father and is carried only by his daughter.

Figure S4. **Result of the linkage analysis based on the STR analysis of chromosome 17 (see Table S3).** The proband is marked with an arrow. Paternal chromosome is in blue and maternal is in red. The mutated *RAD51C* is depicted in blue, wild-type is in pink. Both children carry wild-type *TP53* allele (in purple). The son inherited the paternal grandmother chromosome 17, which carries a wild-type *TP53* allele, thus the *de novo* *TP53* variant occurred in the proband on the paternal chromosome in *cis* with the *RAD51C* variant. For more confidence, we additionally tested the children in regard to the *RAD51C* variant. The recombined chromosome inherited by the daughter carries the variant, while the son harbors none of the variants from the index. Arrows indicate the wild-type *TP53* allele and the mutated *RAD51C* in the daughter.

Figure S5. **Results of loss of heterozygosity (LOH) analysis.** A. Box plot of coverage over the targets in the TruSight Cancer Panel (94 genes, Table see S1). *RAD51C* is an outlier in respect to coverage and the CNV analysis showed a deletion of the wild type allele in tumor. B. Boxplots of the alternative allele frequency (AltAF) of variants present in the TruSight Cancer enriched genes (see Table S1). We do not observe a loss of heterozygosity (LOH) on chromosome 17. Based on the AltAF of the *TP53* variant chr17:7578536; NM_000546.5, c.394A>G, p.(Lys132Glu) we could not conclude that a LOH was present, however in this gene there were only two heterozygous germline variants present. C. p53 immunohistochemical staining of the SGC tumor (2.5 ×). The tumor shows higher p53 expression compared to the surrounding normal tissue. Arrows indicate tumor tissue.

# Supplementary Tables

Table S1. **Genes and single nucleotide polymorphisms (SNPs) targeted in the Illumina TruSight Cancer Sequencing Panel.**

| Gene | Gene | SNP | SNP | SNP | SNP | SNP |
| --- | --- | --- | --- | --- | --- | --- |
| *AIP* | *PTEN* | rs17401966 | rs6939340 | rs10795668 | rs391525 | rs10058728 |
| *ALK* | *RAD51C* | rs9430161 | rs4324798 | rs11012732 | rs258322 | rs872071 |
| *APC* | *RAD51D* | rs7538876 | rs29232 | rs3123078 | rs1805007 | rs4977756 |
| *ATM* | *RB1* | rs11249433 | rs3129055 | rs10993994 | rs4785763 | rs965513 |
| *BAP1* | *RECQL4* | rs7412746 | rs2860580 | rs10821936 | rs4795519 | rs3803662 |
| *BLM* | *RET* | rs3790844 | rs2517713 | rs7089424 | rs4430796 | rs4784227 |
| *BMPR1A* | *RHBDF2* | rs6691170 | rs6457327 | rs10822013 | rs7501939 | rs4793172 |
| *BRCA1* | *RUNX1* | rs6687758 | rs130067 | rs10995190 | rs7210100 | rs242076 |
| *BRCA2* | *SBDS* | rs801114 | rs2894207 | rs224278 | rs1859962 | rs12210050 |
| *BRIP1* | *SDHAF2* | rs1465618 | rs2596542 | rs704010 | rs17674580 | rs4712653 |
| *BUB1B* | *SDHB* | rs7579899 | rs2248462 | rs3765524 | rs7238033 | rs865686 |
| *CDC73* | *SDHC* | rs1432295 | rs3117582 | rs2274223 | rs4939827 | rs505922 |
| *CDH1* | *SDHD* | rs721048 | rs204999 | rs3781264 | rs8170 | rs3112612 |
| *CDK4* | *SLX4* | rs10187424 | rs9268542 | rs17119461 | rs8102137 | rs9929218 |
| *CDKN1C* | *SMAD4* | rs17483466 | rs6903608 | rs12413624 | rs10411210 | rs6603251 |
| *CDKN2A* | *SMARCB1* | rs12621278 | rs2395185 | rs11199874 | rs8102476 | AMG_mid100 |
| *CEBPA* | *STK11* | rs2072590 | rs2858870 | rs2981579 | rs11083846 | MITF_rs149617956 |
| *CEP57* | *SUFU* | rs13016963 | rs674313 | rs2981575 | rs2735839 | ATM_SNP |
| *CHEK2* | *TMEM127* | rs13393577 | rs28421666 | rs1219648 | rs961253 | HOXB13_rs138213197 |
| *CYLD* | *TP53* | rs3768716 | rs2647012 | rs2981582 | rs910873 |  |
| *DDB2* | *TSC1* | rs6435862 | rs10484561 | rs3817198 | rs4925386 |  |
| *DICER1* | *TSC2* | rs13387042 | rs9275572 | rs7127900 | rs6010620 |  |
| *DIS3L2* | *VHL* | rs966423 | rs210138 | rs110419 | rs4809324 |  |
| *EGFR* | *WRN* | rs13397985 | rs10484761 | rs1945213 | rs372883 |  |
| *EPCAM* | *WT1* | rs7584330 | rs339331 | rs11228565 | rs2014300 |  |
| *ERCC2* | *XPA* | rs2292884 | rs2180341 | rs7931342 | rs45430 |  |
| *ERCC3* | *XPC* | rs757978 | rs9485372 | rs10896449 | rs1547374 |  |
| *ERCC4* |  | rs4973768 | rs2046210 | rs7130881 | rs738722 |  |
| *ERCC5* |  | rs1052501 | rs651164 | rs7105934 | rs36600 |  |
| *EXT1* |  | rs2660753 | rs9364554 | rs614367 | rs2284063 |  |
| *EXT2* |  | rs9284813 | rs7758229 | rs1393350 | rs1014971 |  |
| *EZH2* |  | rs17181170 | rs4487645 | rs1801516 | rs5759167 |  |
| *FANCA* |  | rs9841504 | rs11978267 | rs3802842 | rs5768709 |  |
| *FANCB* |  | rs10934853 | rs4132601 | rs498872 | rs1327301 |  |
| *FANCC* |  | rs6763931 | rs6465657 | rs735665 | rs5945572 |  |
| *FANCD2* |  | rs6774494 | rs1495741 | rs2900333 | rs5945619 |  |
| *FANCE* |  | rs10936599 | rs1512268 | rs718314 | rs5919432 |  |
| *FANCF* |  | rs10936632 | rs2439302 | rs10875943 | rs1321311 |  |
| *FANCG* |  | rs4488809 | rs16892766 | rs11169552 | rs3824999 |  |
| *FANCI* |  | rs10937405 | rs1016343 | rs902774 | rs5934683 |  |
| *FANCL* |  | rs17505102 | rs1456315 | rs995030 | rs2283873 |  |
| *FANCM* |  | rs710521 | rs16901979 | rs3782181 | rs807624 |  |
| *FH* |  | rs2131877 | rs2456449 | rs4474514 | rs1027643 |  |
| *FLCN* |  | rs798766 | rs16902094 | rs11066015 | rs3755132 |  |
| *GATA2* |  | rs1494961 | rs445114 | rs671 | rs790356 |  |
| *GPC3* |  | rs12500426 | rs13281615 | rs4767364 | rs5955543 |  |
| *HNF1A* |  | rs17021918 | rs1562430 | rs2074356 | rs10974944 |  |
| *HRAS* |  | rs1229984 | rs10505477 | rs11066280 | rs1210110 |  |
| *KIT* |  | rs971074 | rs6983267 | rs4765623 | rs7555566 |  |
| *MAX* |  | rs7679673 | rs7014346 | rs1572072 | rs1364054 |  |
| *MEN1* |  | rs10069690 | rs1447295 | rs9510787 | rs6734275 |  |
| *MET* |  | rs2242652 | rs4242382 | rs753955 | rs7584993 |  |
| *MLH1* |  | rs2736100 | rs4242384 | rs9600079 | rs17272796 |  |
| *MSH2* |  | rs2853676 | rs7837688 | rs9573163 | rs1155741 |  |
| *MSH6* |  | rs4635969 | rs9642880 | rs9543325 | rs161792 |  |
| *MUTYH* |  | rs4975616 | rs2019960 | rs7335046 | rs11940551 |  |
| *NBN* |  | rs401681 | rs10088218 | rs944289 | rs9293511 |  |
| *NF1* |  | rs31489 | rs891835 | rs116909374 | rs9352613 |  |
| *NF2* |  | rs12653946 | rs4295627 | rs4444235 | rs685449 |  |
| *NSD1* |  | rs2255280 | rs2294008 | rs4779584 | rs7808249 |  |
| *PALB2* |  | rs13361707 | rs7040024 | rs4924410 | rs1106334 |  |
| *PHOX2B* |  | rs2121875 | rs755383 | rs4775302 | rs11017876 |  |
| *PMS1* |  | rs4415084 | rs3814113 | rs8030672 | rs9572094 |  |
| *PMS2* |  | rs889312 | rs7023329 | rs7176508 | rs4905366 |  |
| *PRF1* |  | rs10052657 | rs2157719 | rs8034191 | rs4775699 |  |
| *PRKAR1A* |  | rs20541 | rs1412829 | rs1051730 | rs1528601 |  |
| *PTCH1* |  | rs4624820 | rs1011970 | rs8042374 | rs11655512 |  |

Table S2. **Primers used for the short tandem repeat (STR) analysis of chromosome 17.** Markers that were informative are represented in bold (see Table S3). Fwd = forward, Rev = reverse

| **Locus** | **Dye** | **Primer Name** | **Primer Sequence** | **Average Product Length (bp)** | **Chromosomal coordinates (hg19)** |
| --- | --- | --- | --- | --- | --- |
| D17S949 | FAM | D17S949_FAM_Fwd | CATTTTGAGGATAGAAACTCCA | 109 | chr17:68,465,446-68,465,554 |
|  | FAM | D17S949_FAM_Rev | TTTATCACGGCATTATCAGC |  |  |
| **D17S1852** | FAM | D17S1852_FAM_Fwd | TACAGTTTCTTGTGTGCCC | 202 | chr17:10,515,507-10,515,708 |
|  | FAM | D17S1852_FAM_Rev | AGTAACTCTGAGGACTTGCTCAT |  |  |
| **D17S799** | ROX | D17S799_ROX_Fwd | TGTGACATATTGCCAGCCGTCAG | 195 | chr17:13,170,963-13,171,157 |
|  | ROX | D17S799_ROX_Rev | CAGCATATCATTATAGACAAGCAAAG |  |  |
| **D17S787** | HEX | D17S787_HEX_Fwd | TGGGCTCAACTATATGAACC | 152 | chr17:53,282,084-53,282,235 |
|  | HEX | D17S787_HEX_Rev | TTGATACCTTTTTGAAGGGG |  |  |
| D17S944 | Atto550 | D17S944_Atto550_Fwd | GCCCAGGAGGTTGAGACTT | 216 | chr17:61,436,306-61,436,521 |
|  | Atto550 | D17S944_Atto550_Rev | CCTTCCATAGGAACGGCT |  |  |
| **D17S1876** | Atto550 | D17S1876_Atto550_Fwd | AGCTGCTTCTGCAAAGATG | 104 | chr17:4,345,252-4,345,355 |
|  | Atto550 | D17S1876_Atto550_Rev | TACAAGTCCTGGGCCAC |  |  |
| D17S1791 | FAM | D17S1791_FAM_Fwd | AGCTTTTGGTCAACCTG | 271 | chr17:9,156,457-9,156,727 |
|  | FAM | D17S1791_FAM_Rev | GGGTGGGTGGAGTTAC |  |  |
| **D17S1828** | HEX | D17S1828_HEX_Fwd | TGCACTCACAGATTTGCC | 207 | chr17:3,810,467-3,810,673 |
|  | HEX | D17S1828_HEX_Rev | TTAAGCCAGTTCGGATTTG |  |  |
| D17S957 | ROX | D17S957_ROX_Fwd | CTTTTCTAACCTCCCCTTACC | 144 | chr17:55,473,708-55,473,851 |
|  | ROX | D17S957_ROX_Rev | ACTAGGAATGGGACCACTGT |  |  |

Table S3. **STR analysis results.** For the proband, at informative sites the maternal marker is marked with red, while the paternal is marked with blue. The same representation is maintained for the proband’s children to mark the origin of the chromosome marker (red – paternal grandmother, blue – paternal grandfather)

| **Person/Marker** | **D17S949** | | **D17S1852** | | **D17S1791** | | **D17S787** | | **D17S1828** | | **D17S1876** | | **D17S944** | | **D17S957** | | **D17S799** | |
| --- | --- | --- | --- | --- | --- | --- | --- | --- | --- | --- | --- | --- | --- | --- | --- | --- | --- | --- |
| **Son** | 105 | 105 | 196 | 208 | 265 | 275 | 134 | 136 | 203 | 203 | 105 | 119 | 210 | 225 | 142 | 144 | 195 | 199 |
| **Daughter** | 105 | 105 | 196 | 202 | 265 | 275 | 136 | 138 | 203 | 203 | 105 | 119 | 210 | 225 | 142 | 144 | 189 | 189 |
| **Proband** | 105 | 105 | 202 | 208 | 265 | 265 | 134 | 138 | 203 | 209 | 105 | 113 | 210 | 210 | 144 | 144 | 189 | 199 |
| **Proband’s mother** | 105 | 105 | 200 | 208 | 265 | 275 | 134 | 136 | 203 | 205 | 105 | 117 | 210 | 213 | 140 | 144 | 195 | 199 |

# Supplementary References

1. Kim S, Scheffler K, Halpern AL, Bekritsky MA, Noh E, Kallberg M *et al*: Strelka2: fast and accurate calling of germline and somatic variants. *Nature methods* 2018; **15:** 591-594.

2. Li H: A statistical framework for SNP calling, mutation discovery, association mapping and population genetical parameter estimation from sequencing data. *Bioinformatics (Oxford, England)* 2011; **27:** 2987-2993.

3. Team RC: R: A language and environment for statistical computing 2013.

4. Wickham H, Francois R, Henry L, Müller K: dplyr: A grammar of data manipulation. *R package version 083* 2019; **3**.

5. Wickham H: *ggplot2: elegant graphics for data analysis*. Springer, 2016.

6. Wickham H: Reshaping data with the reshape package. *Journal of statistical software* 2007; **21:** 1-20.

7. Fox J, Weisberg S: *An R companion to applied regression*. Sage Publications, 2018.

8. Karczewski KJ, Francioli LC, Tiao G, Cummings BB, Alföldi J, Wang Q *et al*: Variation across 141,456 human exomes and genomes reveals the spectrum of loss-of-function intolerance across human protein-coding genes. *BioRxiv* 2019**:** 531210.

9. Soussi T, Leroy B, Devir M, Rosenberg S: High prevalence of cancer-associated TP53 variants in the gnomAD database: A word of caution concerning the use of variant filtering. *Human mutation* 2019; **40:** 516-524.

10. Landrum MJ, Lee JM, Riley GR, Jang W, Rubinstein WS, Church DM *et al*: ClinVar: public archive of relationships among sequence variation and human phenotype. *Nucleic acids research* 2014; **42:** D980-985.

11. Goi K, Takagi M, Iwata S, Delia D, Asada M, Donghi R *et al*: DNA damage-associated dysregulation of the cell cycle and apoptosis control in cells with germ-line p53 mutation. *Cancer Res* 1997; **57:** 1895-1902.

12. Kato S, Han SY, Liu W, Otsuka K, Shibata H, Kanamaru R *et al*: Understanding the function-structure and function-mutation relationships of p53 tumor suppressor protein by high-resolution missense mutation analysis. *Proceedings of the National Academy of Sciences of the United States of America* 2003; **100:** 8424-8429.

13. Monti P, Perfumo C, Bisio A, Ciribilli Y, Menichini P, Russo D *et al*: Dominant-negative features of mutant TP53 in germline carriers have limited impact on cancer outcomes. *Molecular cancer research : MCR* 2011; **9:** 271-279.

14. Richards S, Aziz N, Bale S, Bick D, Das S, Gastier-Foster J *et al*: Standards and guidelines for the interpretation of sequence variants: a joint consensus recommendation of the American College of Medical Genetics and Genomics and the Association for Molecular Pathology. *Genetics in medicine : official journal of the American College of Medical Genetics* 2015; **17:** 405-424.

15. Monti P, Ciribilli Y, Jordan J, Menichini P, Umbach DM, Resnick MA *et al*: Transcriptional functionality of germ line p53 mutants influences cancer phenotype. *Clinical cancer research : an official journal of the American Association for Cancer Research* 2007; **13:** 3789-3795.

16. Ng PC, Henikoff S: SIFT: Predicting amino acid changes that affect protein function. *Nucleic acids research* 2003; **31:** 3812-3814.

17. Schwarz JM, Cooper DN, Schuelke M, Seelow D: MutationTaster2: mutation prediction for the deep-sequencing age. *Nature methods* 2014; **11:** 361-362.

18. Carter NJ, Marshall ML, Susswein LR, Zorn KK, Hiraki S, Arvai KJ *et al*: Germline pathogenic variants identified in women with ovarian tumors. *Gynecologic oncology* 2018; **151:** 481-488.

19. Schnurbein G, Hauke J, Wappenschmidt B, Weber-Lassalle N, Engert S, Hellebrand H *et al*: RAD51C deletion screening identifies a recurrent gross deletion in breast cancer and ovarian cancer families. *Breast cancer research : BCR* 2013; **15:** R120.

20. Schubert S, van Luttikhuizen JL, Auber B, Schmidt G, Hofmann W, Penkert J *et al*: The identification of pathogenic variants in BRCA1/2 negative, high risk, hereditary breast and/or ovarian cancer patients: High frequency of FANCM pathogenic variants. *International journal of cancer* 2019; **144:** 2683-2694.

21. Frebourg T, Bajalica Lagercrantz S, Oliveira C, Magenheim R, Evans DG: Guidelines for the Li-Fraumeni and heritable TP53-related cancer syndromes. *European journal of human genetics : EJHG* 2020.

22. Schneider K, Zelley K, Nichols KE, Garber J: Li-Fraumeni Syndrome: *GeneReviews®[Internet]*: University of Washington, Seattle, 2019.

23. Mandelker D, Donoghue M, Talukdar S, Bandlamudi C, Srinivasan P, Vivek M *et al*: Germline-focussed analysis of tumour-only sequencing: recommendations from the ESMO Precision Medicine Working Group. *Annals of oncology : official journal of the European Society for Medical Oncology* 2019; **30:** 1221-1231.

24. Meindl A, Hellebrand H, Wiek C, Erven V, Wappenschmidt B, Niederacher D *et al*: Germline mutations in breast and ovarian cancer pedigrees establish RAD51C as a human cancer susceptibility gene. *Nature genetics* 2010; **42:** 410-414.

25. Osorio A, Endt D, Fernández F, Eirich K, de la Hoya M, Schmutzler R *et al*: Predominance of pathogenic missense variants in the RAD51C gene occurring in breast and ovarian cancer families. *Human molecular genetics* 2012; **21:** 2889-2898.

26. Vogel WH: Li-Fraumeni Syndrome. *Journal of the Advanced Practitioner in Oncology* 2017; **8:** 742.

27. Yang X, Song H, Leslie G, Engel C, Hahnen E, Auber B *et al*: Ovarian and breast cancer risks associated with pathogenic variants in RAD51C and RAD51D. *Journal of the National Cancer Institute* 2020.

28. Kuznetsov SG, Haines DC, Martin BK, Sharan SK: Loss of Rad51c leads to embryonic lethality and modulation of Trp53-dependent tumorigenesis in mice. *Cancer research* 2009; **69:** 863-872.

29. Tumiati M, Munne PM, Edgren H, Eldfors S, Hemmes A, Kuznetsov SG: Rad51c- and Trp53-double-mutant mouse model reveals common features of homologous recombination-deficient breast cancers. *Oncogene* 2016; **35:** 4601-4610.

30. Kuznetsov SG, Haines DC, Martin BK, Sharan SK: Loss of Rad51c leads to embryonic lethality and modulation of Trp53-dependent tumorigenesis in mice. *Cancer research* 2009; **69:** 863-872.

31. Harvey M, Vogel H, Morris D, Bradley A, Bernstein A, Donehower LA: A mutant p53 transgene accelerates tumour development in heterozygous but not nullizygous p53-deficient mice. *Nat Genet* 1995; **9:** 305-311.

1. EDTA = Ethylenediaminetetraacetic acid [↑](#footnote-ref-1)
2. TE Buffer = 10mM Tris Base + 1mM EDTA [↑](#footnote-ref-2)
3. <https://www.qiagen.com/dk/resources/resourcedetail?id=e837c218-433e-4ad8-8ad6-0e5d1bef8b33&lang=en> [↑](#footnote-ref-3)
4. bp = base pairs [↑](#footnote-ref-4)
5. ACMG = American College of Medical Genetics and Genomics [↑](#footnote-ref-5)
